# The Effect of Ultrasound Treatment in Winemaking on the Volatile Compounds of Aglianico, Nero di Troia, and Primitivo Red Wines

**DOI:** 10.3390/foods12030648

**Published:** 2023-02-02

**Authors:** Giuseppe Natrella, Mirella Noviello, Antonio Trani, Michele Faccia, Giuseppe Gambacorta

**Affiliations:** 1 Department of Soil, Plant and Food Science (DISSPA), University of Bari Aldo Moro, Via Amendola 165/A, 70126 Bari, Italy; 2 International Centre for Advanced Mediterranean Agronomic Studies (CIHEAM) of Bari, Via Ceglie 9, 70010 Valenzano, BA, Italy

**Keywords:** Italian grape cultivar, ultrasound maceration, SPME-GC-MS, VOC profile, sustainable technology

## Abstract

An ultrasound (US) treatment was applied during the vinification of three different red grape cultivars with the aim of assessing the impact on the volatile profile of the wines. A robust solid phase micro extraction coupled with gas chromatography mass-spectrometry (SPME-GC-MS) method was developed in order to fix the best parameters for optimizing the volatile organic compound (VOC) recovery. A 15% NaCl solution was added to the samples to increase the salting-out effect, the time/temperature were appropriately selected, and the matrix effect was evaluated by comparing synthetic and real matrices. In addition, external calibration curves were used to quantify the single volatile compounds. The analyses of the wine samples at 7 and 14 months of aging revealed that US exerted the highest effect on Aglianico, which had the highest amount of total VOC. US Nero di Troia showed similar results after 14 months of aging, while Primitivo was not affected by the treatment. Nevertheless, from discriminant analysis, a clear separation was observed between the control and ultrasound-treated wines for all three cultivars, with ethyl decanoate, ethyl isopentyl succinate, and butyric acid having the highest discriminant coefficients. In conclusion, the obtained results indicated that the effect of US treatment on the VOC profile of the wine considered in the experimentation is cultivar-dependent.

## 1. Introduction

Ultrasound (US) technology has been widely employed in the food industry for years. It has numerous advantages: it is a sustainable and low-cost technology with rapid and efficient processes, improving the shelf life and preserving the quality, freshness, taste, nutritional elements, and organoleptic properties of food without wasting energy [1]. This technology is based on the application of mechanical waves of different frequencies (>20 kHz), which are the cause of a phenomenon known as cavitation, wherein produced bubbles collapse and implode, releasing energy, which increases temperature and pressure, causing damages on surfaces [2].

In the food industry, US technology is applied in order to improve processing methods such as gelation or crystallization and to modify the structure and property of foods (i.e., modification of polysaccharides and fats) or to facilitate beneficial food reactions (i.e., glycosylation and enzymatic cross-linking) [3]. Moreover, the literature reports the elimination of pesticides and allergens, and the reduction of microbial contaminants in foods such as milk, fruits, and vegetables [4,5].

With regard to the enological industry, ultrasounds have been explored in several stages of the winemaking process and for different purposes [3], such as to reduce the use of SO_2_ in controlling the microbiological population in wine [6,7,8]. Moreover, this technology could be used to accelerate wine aging on lees. With this aim, different authors have studied their impacts on the polysaccharide release and the organoleptic properties of red wine [9,10,11,12]. The sonication process has also been tested as an artificial aging method in order to improve wine quality, reducing the processing time [13].

Recently, the International Organization of Vine and Wine has approved the use of ultrasound to promote a rapid extraction of grapes’ compounds during pre-fermentation maceration, after destemming and crushing [14]. Thus, the application of US seems to accelerate the winemaking process; indeed, maceration can be reduced from 7 to 3 days [15,16]. In fact, the physical rupture caused by the sonication process degrades the cell walls of grape skins and helps to improve the chromatic characteristics of the final wines, facilitating the extraction of both phenolic and aromatic compounds [15,16,17,18,19,20,21,22]. Ultrasound treatment was applied to many grape cultivars, obtaining different results in terms of volatile profile. Oliver Simancas et al. [20] reported that the use of pilot ultrasound equipment increases the concentration of free varietal volatile compounds such as C6 alcohols, terpenes, and norisoprenoids in Monastrell musts and wines. On the other hand, other authors found a small decrease in the VOC content of Syrah wine after the treatment; nevertheless, the sensory characteristics were improved [23].

To our knowledge, no information is available on the effect of US on the volatile composition of Aglianico, Nero di Troia, and Primitivo wines. The present work aimed to close this gap by determining the effect of ultrasound technology applied during maceration on the volatile profile of wines obtained from these cultivars; with this aim, a suitable protocol for VOC extraction was developed.

## 2. Materials and Methods

### 2.1. Chemicals

All the pure standards of volatile organic compounds (VOC) used in this manuscript were bought from Sigma-Aldrich (Steinheim, Germany): ethyl acetate (99.9%), hexanal (98%), 2-methyl-1-propanol (>99.5%), 1-butanol (99.9%), (E)-2-hexanal (98%), 1-pentanol (>99%), hexyl acetate (99%), 1-hexanol (>99.5%), *cis*-3-hexen-1-ol (98%), ethyl octanoate (>98%), 1-heptanol (>99.5%), linalool (>97%), isobutyric acid (99%), butyric acid (>99%), isovaleric acid (99%), geranyl acetate (>97%), β-citronellol (95%), nerol (98%), phenyl ethyl acetate (98%), β-damascenone (>90%), hexanoic acid (>99%), 2-phenyl ethanol (>99%), and β-ionone (>95%).

### 2.2. Winemaking Process

**Figure 1:**
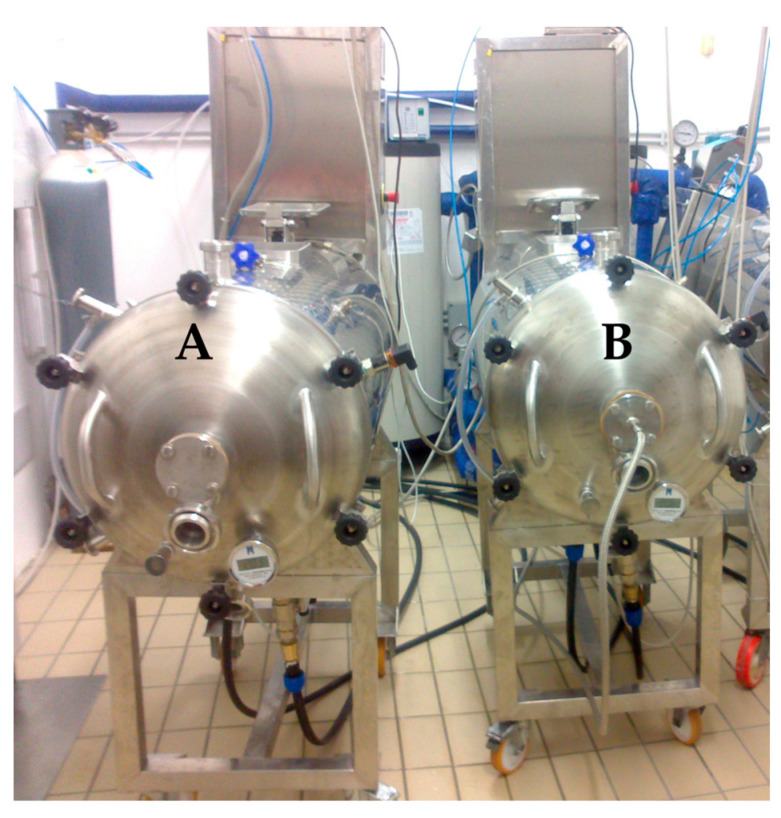
Rotary wine fermenter pilot plants: (**A**) control; (**B**) with ultrasound system.

The winemaking process was carried out according to Gambacorta et al. [22] on three different cultivars of *Vitis vinifera* L.: (i) Primitivo (from Gioia del Colle); (ii) Nero di Troia (from Corato); iii) Aglianico (from Genzano di Lucania). The vineyards involved in the experimentation were situated in Southern Italy, the latter located in the Basilicata region, the other two in the Apulia region. The harvest was carried out considering the optimum grape maturity of each cultivar: Primitivo (19.4 °Brix), Nero di Troia (20.2 °Brix), and Aglianico (21.9 °Brix). Vinification was performed using two different rotary wine fermenter pilot plants of 200 L capacity called “Gioiello” (Industrie Fracchiolla, Adelfia, Italy) as shown in Figure 1; the equipment scheme has been reported in a previous paper [22]. One plant was used to produce the control wines (Ctr, Figure 1A), while the other one was dedicated to the preparation of the ultrasound-treated wines (US, Figure 1B) by equipping it with an ultrasound delivery system (insertion of a transducer along the central axis). The traditional red winemaking process was carried out as follows: grapes were destemmed and crushed, put into the fermenter, and had yeast (20 g 100 kg^−1^ of *Saccharomyces cerevisiae*, var. *Bayanus* Mycoferm^®^, Ever, Pramaggiore, Italy), yeast activator (20 g 100 kg^−1^, Enovit, AEB, Venice, Italy), and potassium metabisulphite (10 g 100 kg^−1^) added. The maceration process lasted 7 days at 25 °C with four rotational cycles every six hours. At the end of the maceration process, the free-run wine was transferred to a 100 L stainless steel vat. The grape pomace was gently pressed to recover the press wine that was added to the free-run wine. At the end of the process, the gross lees were removed, and the wines were bottled and stored at room temperature until analysis at 7 and 14 months after the winemaking process.

The ultrasound wines were produced as reported for the control wines with the following modification: a two-hour ultrasound treatment was applied before the start of maceration at 25 kHz of frequency and 1500 W with 60 W/L of effective power by using an ultrasonic system composed of a Sonic Digital LC 1500 SD 25-P ultrasonic generator and a Sonopush HD Double Twin 1500 Titanium transducer (Weal, Milan, Italy).

### 2.3. VOC Analysis

#### 2.3.1. VOC Extraction

Volatile compound analysis was carried out by using an optimized solid phase micro extraction (SPME) technique. A 50/30 μm divinylbenzene/carboxen/polydimethylsiloxane (Supelco, Bellefonte, PA, USA) fiber was selected as the most suited for the purpose. One milliliter of sample, 10 μL of internal standard solution (2-Octanol, 82 ng), and 0.14 g of NaCl were inserted into a 15 mL glass vial and closed with a screw cap with a perforable septum. Samples were heated up to 50 °C and held for 10 min for the equilibration phase, then the fiber was inserted into the vial headspace for 30 min for the adsorption of volatile compounds. Thereafter, volatiles were desorbed by inserting the fiber into the gas chromatograph (GC) injector for 3 min.

#### 2.3.2. Chromatographic Conditions

VOC analysis was carried out by means of a gas chromatograph (Trace 1300, Thermo Scientific, Waltham, MA, USA) coupled with a mass spectrometer (ISQ, Thermo Scientific). Helium was used as the carrier gas (33 cm s^−1^) and the analytes’ separation was carried out on a TR-WAX MS column (20 m × 0.10 mm I.D., 0.1 μm film thickness, Thermo Scientific). The oven ramp temperature was set as follows: from 50 to 180 °C (ramp rate 13 °C min^−1^), from 180 to 220 °C (ramp rate 18 °C min^−1^), then isothermal conditions for 3 min. The MS settings were: 70 eV of electron impact ionization, mass acquisition range 33–280 amu, acquisition rate of 7.2 Hz. The chromatograms were acquired in total ion current, full scan mode. Compound identification was performed by comparing mass spectra with those present in the National Institute of Standards & Technology (NIST) library or by standard when available. Quantitative analysis was carried out using the external standard method. Indeed, the calibration curves of pure standards (at concentrations ranging from 0.01 to 0.8 mg L^−1^) listed in Section 2.1 were developed by using a synthetic matrix (14% ethanol, 5 g L^−1^ tartaric acid, and 3.3 pH buffer); an internal standard widely used in the literature (2-octanol, 8.2 mg L^−1^) [24] was used to normalize the standard area variability. Finally, limit of detection (LOD) and limit of quantification (LOQ) were calculated and listed in Table S1 of the Supplementary Material. The quantification was carried out using these curves, but when compounds were identified with the NIST library (without specific standard), they were indirectly quantified using the calibration curve of the analogue compound as reported in the literature (i.e., hexyl acetate for the quantification of 3-methyl-1-butyl acetate and nerol for geraniol) [25].

### 2.4. Statistical Analysis

The data collected from the method developing phase and the wine results were analyzed statistically using IBM SPSS software (IBM Corp., Armonk, NY, USA). Analysis of variance (ANOVA) of the means was calculated to evaluate the effect of US treatment and to highlight the differences between the cultivars. Tukey’s HSD test was used to observe differences between the cultivars that were independent from the treatment. Finally, canonical discriminant functions analysis was performed on both independent variables “cultivar” and “treatment” to synthetize results and maximize the existing differences between the samples. Each analysis was carried out in triplicates.

## 3. Results and Discussion

### 3.1. Optimization of VOC Extraction Procedure

The choice of the fiber is a crucial point to obtain reliable results, and it is well known that no fiber coating is able to extract all analytes with the same efficiency. In our case, since an untargeted extraction was needed, a 50/30 μm divinylbenzene/carboxen/polydimethylsiloxane fiber was selected, being able to adsorb a wide range of molecules with different characteristics due to the presence of three different coatings. In addition to this, the selected fiber was particularly suited for wine volatiles’ adsorption, as reported in the literature [26,27,28,29,30].

Concerning the temperature of extraction, this is strictly related to the volatility of the molecules, some of which volatilize slowly at room temperature. As a matter of fact, temperature has an important effect on the kinetics of the extraction process, influencing the vapor pressure of molecules. Consequently, different extraction temperatures were tested (30, 40, and 50 °C). It was found that the extraction of semi-volatile compounds was only obtained when operating at high temperatures, whereas the low boiling ones were extracted at all temperatures (even though the effectiveness decreased as the temperature increased). Finally, 50 °C was the chosen temperature, because it allowed a satisfactory recovery of both groups of volatiles (data not shown).

**Figure 2:**
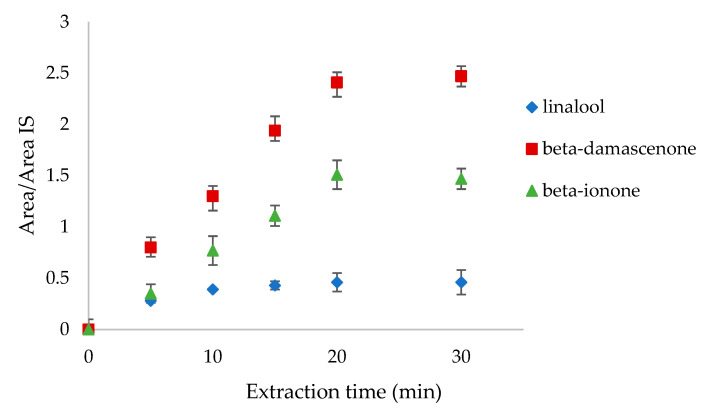
Extraction time tests on linalool, β-damascenone, and β-ionone at 5, 10, 15, 20, and 30 min.

In terms of the extraction time, 5, 10, 15, 20, and 30 min were tested analyzing the headspace of linalool, β-damascenone, and β-ionone mixture standards at 0.1 mg L^−1^ concentration (Figure 2). The suitable extraction time is when the amount of the extracted compounds remains constant over time. In our case, linalool reached equilibrium after 20 min, whereas the other two compounds reached it after 30 min. In order to include all the volatiles, the equilibration time of 30 min was chosen.

The addition of two different NaCl concentrations (15 and 30%) was tested to improve the extraction of VOC as a consequence of the salting-out effect. In our case, the highest extraction efficiency was found when 15% salt was used, likely due to the fact that higher salt concentrations tend to generate electrostatic interactions that negatively affect the extraction process [31,32].

**Figure 3:**
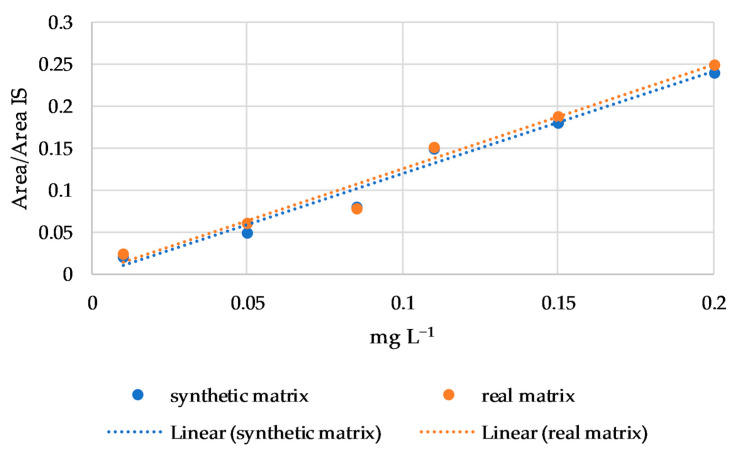
Regression lines of nerol in synthetic and real matrix.

Finally, the matrix effect was evaluated using regression lines of linalool, nerol, β-citronellol, and β-damascenone in a synthetic and in a real matrix. These four compounds were chosen for their wide hydrophobicity range (from 2.97 to 4.29 log P) and because they are subject to a matrix effect in connection with the cultivar, as reported by Rodríguez-Bencomo et al. [33] in a study on young Beaujolais and oak-aged Tempranillo red wines. No differences were found in the regression line slopes between the synthetic and real matrix for all four compounds, as shown for nerol in Figure 3. Therefore, according to the Student’s t test, no matrix effect was observed.

### 3.2. Effect of US Treatment on the VOC Profile

Overall, 34 compounds were identified in the headspace of the wine samples, all already reported in the literature [34,35,36,37]: 10 esters, 8 alcohols, 6 acids, 5 terpenes, 2 aldehydes, 2 norisoprenoid, and 1 thiol. Table 1 shows the details of their identification, including retention time (RT, min), quantifier ion (Qt, *m*/*z*), qualifier ion (Qi, *m*/*z*), and odor threshold, along with odor descriptor.

Table 2 shows the VOC profile of the 7-month-aged wine samples. Eleven compounds were under the limit of quantification for all the cultivar in both Ctr and US samples. 2+3-methyl butanol had the highest concentration in all samples, ranging from ≈71 to 79 mg L^−1^ in Ctr and from ≈66 to 83 mg L^−1^ in US. Ethyl acetate was the second-highest VOC found, ranging from ≈32 to 49 mg L^−1^ in Ctr and from ≈31 to 52 mg L^−1^ in US. Considering each cultivar separately, some differences were found between treated and untreated samples: ethyl acetate, 2-methyl-1-propanol, 2+3-methyl butanol and 2-phenyl ethanol were higher in Aglianico US wine than Ctr; on the other hand, Ctr Nero di Troia wine had a higher amount of 2+3-methyl butanol, (E)-2-hexenal, acetic acid, hexanoic acid, ethyl isopentyl succinate, and 2-phenyl ethanol than US. Finally, Primitivo US wine had a statistically higher amount of 2-methyl-1-propanol, diethyl succinate, and octanoic acid, but lower content of acetic acid than Ctr. According to the scientific literature, ultrasound treatment increases the extraction of thiols, terpenes, and C6 alcohols [20]. In the present experimentation, only 3-methylthio-1-propanol was higher in the US-treated Aglianico sample than in the control, whereas an opposite trend was observed for 1-hexanol, which was higher only in the Nero di Troia control sample. In addition, no effect on terpenes was shown, since most of them were under the limit of quantification. The literature reports uneven opinions on the effect of US on esters: some authors reported a lower content (attributable to a degassing effect) or non-significant changes after the treatment [15,20], while others observed an increasing trend [60,61]. Our results allow us to hypothesize a cultivar-dependent behavior, since a higher amount of ethyl acetate was found in the Aglianico US sample than Ctr, but not in the other two cultivars, ethyl octanoate was higher in the US Nero di Troia and Primitivo samples, and diethyl succinate was higher in the US samples for all cultivars. The Aglianico US sample also had a higher level of alcohols, in agreement with other authors [15].

In general, the release of volatile compounds from grape skin depends on the permeability of the cell walls, as well as on the ultrasound treatment conditions (laboratory probes, frequency, and time), which determines the extent of skin breaking. In our case, since the parameters of the US treatment were the same, the cultivar played an important role, showing Aglianico as the most inclined to release VOC after the treatment. In fact, when considering the total concentrations, US treatment had a positive impact only on this cultivar, while an opposite trend was observed for Nero di Troia and there was no effect for Primitivo. It can be concluded that the effect of US treatment is cultivar-dependent, as previously hypothesized [21,22].

When considering the odor activity values, all the samples shared the same 13 volatile compounds being theoretically aroma-active. The molecules were ethyl acetate, 2+3-methyl butanol, ethyl hexanoate, ethyl octanoate, linalool, 3-methylthio-1-propanol, hexanoic acid, 2-phenyl ethanol, 3-methyl-1-butyl acetate, (E)-2-hexenal, butyric acid, isovaleric acid, and octanoic acid. They could theoretically be responsible for different odor perception according to their concentration: in general, they confer fruity, green, sweet, floral, cooked vegetables, fatty–rancid, cheesy, and fatty odor notes.

Table 3 shows the concentrations of the VOC found after 14 months of aging. As already observed for 7-month-aged wines, the US effect seemed to be cultivar-dependent [15,60]. Again, a remarkable impact was observed for Aglianico, followed by Nero di Troia, whereas a scarce effect was found in Primitivo. In particular, ethyl acetate was found at a concentration of ≈114–108 mg L^−1^ (US Aglianico and Nero di Troia) vs. ≈65–95 mg L^−1^ (Ctr Aglianico and Nero di Troia); an increasing trend for this compound was already reported in the literature [11]. Several alcohols were more abundant in the US than Ctr wine samples (2+3-methyl butanol, 1-hexanol and 2-phenyl ethanol). Bautista-Ortin et al. [15] found slightly higher amounts of alcohols in US samples (though not at significant levels). Many other VOC were statistically higher in the US Aglianico and Nero di Troia than Ctr samples, such as (E)-2-hexenal (probably extracted by the cavitation phenomena on grape skin), acetic acid, isobutyric acid, butyric acid, ethyl decanoate, diethyl succinate, and octanoic acid. On the other hand, only a few volatiles were higher in the Primitivo US than Ctr, such as acetic acid, butyric acid, diethyl succinate, and hexanoic acid. Similar compounds across the wine cultivars had different trends, thus confirming the influence of the cultivar during the ultrasound treatment. It is important to emphasize that after 14 months of aging, the US Nero di Troia sample shared many compounds with US Aglianico that were more abundant than in the Ctr. This result could indicate a late influence of the US treatment, which could trigger the beginning of reactions leading to the formation of volatile compounds or could release precursors from grape skin that became volatile later [20].

Considering the total VOC, similar conclusions can be drawn: US Aglianico wine had a concentration of 449.94 vs. 303.60 mg L^−1^ in Ctr and a similar behavior was found in Nero di Troia (422.91 in US and 339.17 mg L^−1^ in Ctr); the opposite was found in Primitivo, with 393.41 mg L^−1^ in Ctr vs. 361.59 mg L^−1^ in US. The absence of effect of US on this latter cultivar could be connected with the morphological characteristics of the grape, which presents a very thin skin that is easily disrupted under normal conditions and, consequently, cannot take advantage of the US treatment. Regarding the possible impact on aroma perception, after 14 months of wine aging, the compounds exceeding the odor threshold had a total concentration of 17.4 higher than in the 7-month-aged wine. The odor-active compounds were similar to those found after 7 months of aging (Table 2) except for ethyl decanoate, *cis*-3-hexen-1-ol, 2-methyl-1-propanol, and isobutyric acid. Ethyl decanoate was aroma-active (odor activity value, OAV > 1) in Aglianico and Nero di Troia; *cis*-3-hexen-1-ol had an OAV of 1.1 and 1.4 in Ctr and US Nero di Troia, respectively; 2-methyl-1-propanol had an OAV of 1 in US and Ctr Primitivo samples, and, finally, isobutyric acid had the highest values in the Aglianico and Nero di Troia US samples. These compounds supply fruity, brandy, alcoholic, flower, and fatty–rancid odor notes.

**Figure 4:**
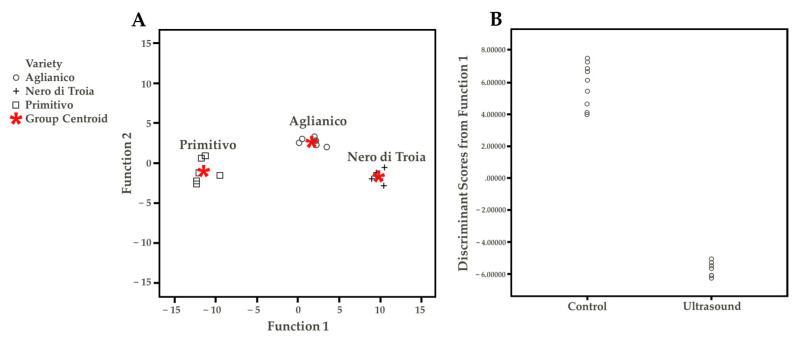
(**A**) Discriminant function plot considering wine cultivar. (**B**) Discriminant function plot considering the treatment.

The Tukey HSD (honestly significant difference) test allowed determining which VOC discriminated the wine cultivars, independent from the US treatment (Table S2). Under the technological conditions of this study, the possible discriminant compounds were ethyl hexanoate, ethyl decanoate, ethyl isopentyl succinate, and butyric acid for Nero di Troia, diethyl succinate and 1-hexanol for Primitivo, and linalool and *cis*-3-hexen-1-ol for Aglianico. These differences were probably responsible for the results of the discriminant function plot (Figure 4A). This statistical approach aimed to summarize the quantitative VOC results. The discriminant function analysis maximizes the existing differences between groups. Wine cultivar (three groups: Aglianico, Nero di Troia, Primitivo) and treatment (two groups: Ctr and US) were used as variables. Based on the differences found in the Tukey test, Figure 4A shows, within the two-discriminant function, a clear separation of the wine cultivar, demonstrating the “concrete differences” in the VOC profile between the samples. Considering the treatment factor (Figure 4B), only one discriminant function was obtained, in which it is possible to observe the separation of the control and treated samples. The single VOC found with the highest discriminant coefficient were ethyl decanoate, ethyl isopentyl succinate, and butyric acid (Table S3). To conclude, the volatile profile was deeply influenced by both the cultivar and US treatment applied.

## 4. Conclusions

To conclude, this investigation allowed the development of a suitable SPME-GC/MS method to characterize the VOC profile of Primitivo, Nero di Troia, and Aglianico wines obtained with or without ultrasound treatment. This sustainable technology had an influence on the VOC profile of the wines to different extents, depending on the cultivar considered. Aglianico samples was the most inclined to release VOC from grapes after the ultrasound treatment, showing higher volatile compound content than the control both after 7 and 14 months of aging. A late effect of ultrasound treatment was observed for Nero di Troia, which shared many VOC significantly higher than the control after 14 months with the US Aglianico sample. Finally, Primitivo showed some changes in volatile profile but to a lesser extent than the other cultivars. Unfortunately, we were not able to determine if these changes had an impact on the sensory point of view. Further study is needed, with the help of a highly qualified panel, to determine if the consequences are positive or not in terms of aroma perception.

## Figures and Tables

**Table 1:** Retention time (RT), quantifier ion (Qt), qualifier ion (Qi), odor threshold (mg L^−1^), and the odor descriptor of the identified VOC in wine samples.

NAME	RT (min)	Qt	Qi	Odor Threshold (mg L^−1^)	Odor Descriptor
ethyl acetate	1.68	61	73	12.26 [38]	Pineapple, Fruity, Solvent, Balsamic
hexanal	2.77	56	57	0.1 [39]	Grassy, Green
2-methyl-1-propanol	2.86	42	74	40 [40]	Alcohol, Solvent
3-methyl-1-butyl acetate	3.07	43	70	0.002 [41]	Banana, Fruity, Sweet
1-butanol	3.28	56	41	150 [42]	Medicinal
2+3-methyl butanol	3.83	55	57	30 [43]	Alcohol, Sweet, Fruity
(E)-2-hexenal	3.99	69	83	0.017 [44]	Green, Fresh, Fruity
ethyl hexanoate	4.08	88	99	0.004 [45]	Fruity, Green, Apple, Banana, Brandy
1-pentanol	4.24	42	55	4 [46]	Almond, Synthetic, Balsamic
hexyl acetate	4.46	56	69	0.01 [47]	Green, Floral
1-hexanol	5.24	56	55	2.5 [44]	Herbaceous, Grassy, Woody
*cis*-3-hexen-1-ol	5.58	67	41	0.07 [48]	Plant, Flower
ethyl octanoate	6.06	88	101	0.0016 [45]	Sweet, Fruity, Banana, Pear, Brandy
1-heptanol	6.25	70	55	1 [49]	Oily
acetic acid	6.3	43	60	200 [50]	Vinegar
linalool	7.13	71	93	0.006 [51]	Citrus, Floral, Sweet
isobutyric acid	7.42	43	73	8.1 [52]	Fatty, Rancid
butyric acid	7.98	60	73	0.24 [52]	Fatty–Rancid, Sweat, Cheese
ethyl decanoate	7.99	88	101	0.2 [45]	Brandy, Fruity, Grape
isovaleric acid	8.34	60	73	0.12 [52]	Fatty–Rancid, Cheese
diethyl succinate	8.35	101	129	200 [53]	Fruity, Melon
3-methylthio-1-propanol	8.76	61	106	0.5 [54]	Cooked vegetable, Baked cabbage
geranyl acetate	9.05	69	93	0.06 [55]	Floral
β -citronellol	9.13	69	95	0.1 [55]	Green, Lemon
geraniol	9.33	69	123	0.03 [55]	Citric, Floral
nerol	9.44	69	41	0.5 [55]	Rose
phenyl ethyl acetate	9.61	105	43	0.25 [55]	Pleasant, Floral
β-damascenone	9.68	69	105	0.00005 [55]	Sweet, Fruity
hexanoic acid	9.85	60	73	0.42 [56]	Cheese, Fatty
ethyl isopentyl succinate	10.27	101	129		
2-phenyl ethanol	10.41	91	122	1.1 [57]	Rose, Honey
β-ionone	10.62	177	91	0.0035 [58]	Balsamic, Rose, Violet
octanoic acid	11.46	60	73	0.5 [59]	Fatty, Rancid
monoethyl succinate	13.59	101	73		Chocolate

**Table 2:** Single volatile compound identified and quantified in control and ultrasound-treated wine samples after 7 months of aging (mg L^−1^).

	Cultivar						OAV					
	Aglianico		Nero di Troia		Primitivo		Aglianico		Nero di Troia		Primitivo	
	Ctr	US	Ctr	US	Ctr	US	Ctr	US	Ctr	US	Ctr	US
ethyl acetate	^†^ 34.51 ± 0.81 b	44.71 ± 1.05 a	32.64 ± 0.76	31.39 ± 0.74	49.23 ± 1.16	52.68 ± 1.24	2.8	3.6	2.7	2.6	4.0	4.3
hexanal	<LOQ	<LOQ	<LOQ	<LOQ	<LOQ	<LOQ						
2-methyl-1-propanol	14.10 ± 0.28 b	23.95 ± 0.48 a	15.89 ± 0.37 b	18.73 ± 0.37 a	16.83 ± 0.34 b	24.45 ± 0.49 a	0.4	0.6	0.4	0.5	0.4	0.6
3-methyl-1-butyl acetate *	0.17 ± 0.01	0.20 ± 0.02	0.11 ± 0.01 b	0.28 ± 0.01 a	0.15 ± 0.01	0.18 ± 0.02	85	100	55	140	75	90
1-butanol	0.75 ± 0.02 b	0.95 ± 0.03 a	0.62 ± 0.02 a	0.48 ± 0.02 b	0.79 ± 0.02 a	0.68 ± 0.02 b	0.0	0.0	0.0	0.0	0.0	0.0
2+3-methyl butanol	79.43 ± 1.79 b	83.48 ± 1.88 a	71.74 ± 1.62 a	66.37 ± 1.50 b	79.05 ± 1.78 a	73.27 ± 1.65 b	2.6	2.8	2.4	2.2	2.6	2.4
(E)-2-hexenal	0.06 ± 0.01	0.04 ± 0.01	0.96 ± 0.05 a	0.04 ± 0.01 b	0.03 ± 0.01	0.05 ± 0.01	3.5	2.4	56.5	2.4	1.8	2.9
ethyl hexanoate *	0.07 ± 0.01	0.07 ± 0.01	0.08 ± 0.01	0.08 ± 0.01	0.06 ± 0.01	0.08 ± 0.01	17.5	17.5	20	20	15	20.3
1-pentanol	<LOQ	<LOQ	<LOQ	<LOQ	<LOQ	<LOQ						
hexyl acetate	<LOQ	<LOQ	<LOQ	<LOQ	<LOQ	<LOQ						
1-hexanol	0.51 ± 0.01	0.52 ± 0.01	0.57 ± 0.01 a	0.49 ± 0.01 b	0.40 ± 0.01	0.40 ± 0.01	0.2	0.2	0.2	0.2	0.2	0.2
*cis*-3-hexen-1-ol	0.03 ± 0.01	0.03 ± 0.01	0.06 ± 0.01	0.05 ± 0.01	0.02 ± 0.01	0.02 ± 0.01	0.4	0.4	0.9	0.7	0.3	0.3
ethyl octanoate	0.28 ± 0.01	0.27 ± 0.01	0.16 ± 0.01 b	0.34 ± 0.01 a	0.28 ± 0.01 b	0.34 ± 0.01 a	175	169	100	212.5	175	212.5
1-heptanol	<LOQ	<LOQ	<LOQ	<LOQ	<LOQ	<LOQ						
acetic acid *	3.59 ± 0.04 b	3.99 ± 0.05 a	6.32 ± 0.01 a	2.59 ± 0.03 b	6.71 ± 0.08 a	5.49 ± 0.07 b	0.0	0.0	0.0	0.0	0.0	0.0
linalool	0.03 ± 0.01	0.06 ± 0.02	0.04 ± 0.01	0.05 ± 0.01	0.05 ± 0.01	0.05 ± 0.01	5.0	10	6.7	8.3	8.3	8.3
isobutyric acid	3.39 ± 0.13 b	4.47 ± 0.17 a	3.50 ± 0.13 b	4.18 ± 0.16 a	6.34 ± 0.24	6.34 ± 0.24	0.4	0.6	0.4	0.5	0.8	0.8
butyric acid	7.98 ± 0.24 b	10.39 ± 0.33 a	6.96 ± 0.21	6.99 ± 0.21	7.41 ± 0.23	7.90 ± 0.28	33.3	43.3	29.0	29.1	30.9	32.9
ethyl decanoate *	0.14 ± 0.02	0.18 ± 0.02	0.12 ± 0.01	0.14 ± 0.01	0.12 ± 0.01	0.13 ± 0.01	0.7	0.9	0.6	0.7	0.6	0.7
isovaleric acid	1.16 ± 0.03 b	1.41 ± 0.03 a	0.84 ± 0.03 a	0.61 ± 0.02 b	1.65 ± 0.04 b	2.11 ± 0.05 a	4.8	5.9	3.5	2.5	6.9	8.8
diethyl succinate *	0.67 ± 0.03 b	0.93 ± 0.04 a	0.19 ± 0.01 b	0.45 ± 0.02 a	1.29 ± 0.05 b	2.50 ± 0.11 a	0.0	0.0	0.0	0.0	0.0	0.0
3-methylthio-1-propanol *	1.91 ± 0.04 b	2.17 ± 0.04 a	1.52 ± 0.03 a	1.08 ± 0.03 b	1.76 ± 0.04 a	1.61 ± 0.03 b	3.8	4.3	3.0	2.2	3.5	3.2
geranyl acetate	<LOQ	<LOQ	<LOQ	<LOQ	<LOQ	<LOQ						
β-citronellol	<LOQ	<LOQ	<LOQ	<LOQ	<LOQ	<LOQ						
geraniol *	<LOQ	<LOQ	<LOQ	<LOQ	<LOQ	<LOQ						
nerol	<LOQ	<LOQ	<LOQ	<LOQ	<LOQ	<LOQ						
phenyl ethyl acetate	<LOQ	<LOQ	<LOQ	<LOQ	<LOQ	<LOQ						
β-damascenone	<LOQ	<LOQ	<LOQ	<LOQ	<LOQ	<LOQ						
hexanoic acid	1.34 ± 0.02 b	1.41 ± 0.01 a	1.41 ± 0.02 a	0.85 ± 0.02 b	1.16 ± 0.02 b	1.68 ± 0.02 a	3.2	3.4	3.4	2.0	2.8	4.0
ethyl isopentyl succinate *	0.08 ± 0.01	0.11 ± 0.02	0.47 ± 0.15 a	0.05 ± 0.01 b	0.12 ± 0.01	0.15 ± 0.02						
2-phenyl ethanol	53.71 ± 1.92 b	68.36 ± 2.44 a	47.61 ± 1.70 a	38.09 ± 1.36 b	53.43 ± 1.91	55.59 ± 1.99	48.8	62.1	43.3	34.6	48.6	50.5
β-ionone	<LOQ	<LOQ	<LOQ	<LOQ	<LOQ	<LOQ						
octanoic acid *	4.52 ± 0.05 a	3.44 ± 0.04 b	4.28 ± 0.05 a	3.01 ± 0.04 b	3.91 ± 0.05 b	5.39 ± 0.06 a	9.0	6.9	8.6	6.0	7.8	10.8
monoethyl succinate *	0.16 ± 0.01	0.17 ± 0.01	0.03 ± 0.01	0.05 ± 0.01	0.14 ± 0.01 b	0.30 ± 0.01 a						
Total	208.59 ± 8.41 b	251.31 ± 9.01 a	196.12 ± 5.28 a	176.39 ± 9.64 b	230.93 ± 10.04	241.39 ± 9.07						

^†^ In columns, for each variety, different letters indicate statistically significant differences at *p* < 0.05. * Concentration indirectly estimated using the calibration curve of the analogue compound.

**Table 3:** Single volatile compounds identified and quantified in control and ultrasound-treated wine samples after 14 months of aging (mg L^−1^).

	Cultivar						OAV					
	Aglianico		Nero di Troia		Primitivo		Aglianico		Nero di Troia		Primitivo	
	Ctr	US	Ctr	US	Ctr	US	Ctr	US	Ctr	US	Ctr	US
ethyl acetate	^†^ 65.10 ± 1.53 b	114.42 ± 2.69 a	95.94 ± 2.26 b	108.16 ± 2.54 a	118.87 ± 2.80 a	101.85 ± 2.40 b	5.3	9.3	7.8	8.8	9.7	8.3
hexanal	<LOQ	<LOQ	<LOQ	<LOQ	<LOQ	<LOQ						
2-methyl-1-propanol	28.28 ± 0.57	28.41 ± 0.58	26.04 ± 0.53	22.92 ± 0.47	41.39 ± 0.84 a	38.93 ± 0.78 b	0.7	0.7	0.7	0.6	1.0	1.0
3-methyl-1-butyl acetate *	0.19 ± 0.01	0.20 ± 0.01	0.18 ± 0.01	0.23 ± 0.01	0.20 ± 0.14	0.18 ± 0.01	95	100	90	115	100	90
1-butanol	1.12 ± 0.04	1.32 ± 0.05	1.09 ± 0.04	0.84 ± 0.06	1.40 ± 0.05	1.18 ± 0.03	0.0	0.0	0.0	0.0	0.0	0.0
2+3 methyl butanol	106.07 ± 2.39 b	134.21 ± 3.03 a	103.29 ± 2.33 b	125.87 ± 2.84 a	122.85 ± 2.77 a	104.44 ± 2.36 b	3.5	4.5	3.4	4.2	4.1	3.5
(E)-2-hexenal	0.06 ± 0.01 b	0.98 ± 0.05 a	0.87 ± 0.04 b	1.78 ± 0.08 a	0.06 ± 0.01 b	0.09 ± 0.01 a	3.5	57.6	51.2	104.7	3.5	5.3
ethyl hexanoate *	0.10 ± 0.01	0.10 ± 0.01	0.13 ± 0.01	0.12 ± 0.01	0.10 ± 0.01	0.10 ± 0.01	25	25	32.5	30	25	25
1-pentanol	<LOQ	<LOQ	<LOQ	<LOQ	<LOQ	<LOQ						
hexyl acetate	<LOQ	<LOQ	<LOQ	<LOQ	<LOQ	<LOQ						
1-hexanol	0.92 ± 0.02 b	1.12 ± 0.02 a	1.04 ± 0.02 b	1.10 ± 0.02 a	0.71 ± 0.02	0.72 ± 0.01	0.4	0.4	0.4	0.4	0.3	0.3
*cis*-3-hexen-1-ol	0.05 ± 0.01	0.06 ± 0.01	0.08 ± 0.01	0.10 ± 0.01	0.02 ± 0.01	0.03 ± 0.00	0.7	0.9	1.1	1.4	0.3	0.4
ethyl octanoate	0.38 ± 0.02	0.42 ± 0.02	0.54 ± 0.03 a	0.30 ± 0.02 b	0.41 ± 0.02	0.41 ± 0.02	237.5	262.5	337.5	187.5	256.3	256.3
1-heptanol	<LOQ	<LOQ	<LOQ	<LOQ	<LOQ	<LOQ						
acetic acid *	3.84 ± 0.05 b	11.48 ± 0.14 a	7.17 ± 0.09 b	10.87 ± 0.13 a	6.60 ± 0.08 b	9.07 ± 0.11 a	0.0	0.1	0.0	0.1	0.0	0.0
linalool	0.012 ± 0.001	0.018 ± 0.001	0.007 ± 0.001	0.012 ± 0.001	0.009 ± 0.001	0.012 ± 0.001	2.0	3.0	1.2	2.0	1.5	2.0
isobutyric acid	10.10 ± 0.50 b	13.95 ± 0.53 a	11.49 ± 0.44 b	13.00 ± 0.49 a	13.76 ± 0.53 a	12.13 ± 0.46 b	1.2	1.7	1.4	1.6	1.7	1.5
butyric acid	8.88 ± 0.30 b	12.47 ± 0.40 a	10.61 ± 0.34 b	13.58 ± 0.43 a	7.58 ± 0.26 b	8.63 ± 0.27 a	37	52	44.2	56.6	31.6	36
ethyl decanoate *	0.14 ± 0.02 b	0.20 ± 0.01 a	0.16 ± 0.01 b	0.21 ± 0.01 a	0.12 ± 0.01	0.13 ± 0.01	0.7	1.0	0.8	1.1	0.6	0.7
isovaleric acid	<LOQ	3.38 ± 0.08	2.00 ± 0.05 b	2.76 ± 0.07 a	3.01 ± 0.08	3.24 ± 0.08	0.0	28.2	16.7	23	25.1	27
diethyl succinate *	1.45 ± 0.06 b	2.24 ± 0.09 a	1.28 ± 0.05 b	2.09 ± 0.09 a	2.70 ± 0.11 b	3.22 ± 0.14 a	0.0	0.0	0.0	0.0	0.0	0.0
3-methylthio-1-propanol *	3.02 ± 0.05 b	4.37 ± 0.09 a	2.53 ± 0.05 b	3.52 ± 0.07 a	2.85 ± 0.06 a	2.39 ± 0.05 b	6.0	8.7	5.1	7.0	5.7	4.8
geranyl acetate	<LOQ	<LOQ	<LOQ	<LOQ	<LOQ	<LOQ						
β -citronellol	<LOQ	<LOQ	<LOQ	<LOQ	<LOQ	<LOQ						
geraniol	<LOQ	<LOQ	<LOQ	<LOQ	<LOQ	<LOQ						
nerol	<LOQ	<LOQ	<LOQ	<LOQ	<LOQ	<LOQ						
phenyl ethyl acetate	<LOQ	<LOQ	<LOQ	<LOQ	<LOQ	<LOQ						
β -damascenone	<LOQ	<LOQ	<LOQ	<LOQ	<LOQ	<LOQ						
hexanoic acid	2.14 ± 0.03	2.01 ± 0.04	2.77 ± 0.04 b	3.31 ± 0.04 a	1.95 ± 0.03 b	2.35 ± 0.03 a	5.1	4.8	6.6	7.9	4.6	5.6
ethyl isopentyl succinate *	0.09 ± 0.01 b	0.15 ± 0.01 a	0.06 ± 0.02 b	0.13 ± 0.14 a	0.14 ± 0.03	0.14 ± 0.01						
2-phenyl ethanol *	65.94 ± 2.36 b	108.11 ± 3.86 a	66.26 ± 2.37 b	104.52 ± 3.74 a	69.37 ± 2.48 a	64.85 ± 2.32 b	59.9	98.3	60.2	95	63.1	59
β-ionone	<LOQ	<LOQ	<LOQ	<LOQ	<LOQ	<LOQ						
octanoic acid	5.52 ± 0.07 b	9.97 ± 0.12 a	5.43 ± 0.21 b	7.23 ± 0.10 a	8.99 ± 0.11 a	7.24 ± 0.09 b	11	19.9	11	14.5	18.0	14.5
monoethyl succinate *	0.20 ± 0.01 b	0.35 ± 0.02 a	0.20 ± 0.08	0.26 ± 0.02	0.32 ± 0.02 a	0.26 ± 0.01 b						
Total	303.60 ± 12.01 b	449.94 ± 15.20 a	339.17 ± 17.08 b	422.91 ± 10.51 a	393.41 ± 21.00 a	361.59 ± 18.63 b						

^†^ In columns, for each variety, different letters indicate statistically significant differences at *p* < 0.05. * Concentration indirectly estimated using the calibration curve of the analogue compound.

## Data Availability

The data are available from the corresponding author.

## Supplementary Materials

The following supporting information can be downloaded at: https://www.mdpi.com/article/10.3390/foods12030648/s1, Table S1: LOD and LOQ values (mg L-1) of the external calibration curves; Table S2: Multiple comparison test (Tukey HSD test) results considering the cultivar as independent variable; Table S3: Standardized Canonical Discriminant Function Coefficients of 14-months aged Ctr and US VOC profile of wines.
