# Supplementary material for: The Effect of Ultrasound Treatment in Winemaking on the Volatile Compounds of Aglianico, Nero di Troia, and Primitivo Red Wines
Source: Foods. 2023 Feb 2;12(3):648. doi: 10.3390/foods12030648 (PMC9914304; doi:10.3390/foods12030648)
Supplement: Supplementary file 1 [file foods-12-00648-s001.zip › foods-2144521-supplementary.pdf]

Table S1. LOD and LOQ values (mg L^{-1}) of the external calibration curves.

	Compound	LOD	LOQ
**1**	ethyl acetate	0.022	0.074
**2**	hexanal	0.005	0.017
**3**	2-methyl-1-propanol	0.023	0.076
**4**	1-butanol	0.018	0.060
**5**	2+3 methyl butanol	0.006	0.205
**6**	(E)-2-hexenal	0.010	0.033
**7**	1-pentanol	0.616	2.052
**8**	hexyl acetate	0.007	0.025
**9**	1-hexanol	0.003	0.010
**10**	cis-3-hexen-1-olo	0.004	0.015
**11**	ethyl octanoate	0.009	0.029
**12**	1-heptanol	0.006	0.020
**13**	linalool	0.001	0.004
**14**	isobutyric acid	0.060	0.200
**15**	butyric acid	0.090	0.300
**16**	isovaleric acid	0.020	0.050
**17**	geranyl acetate	0.008	0.030
**18**	β-citronellol	0.004	0.012
**19**	nerol	0.005	0.020
**20**	phenyl ethyl acetate	0.020	0.070
**21**	β -damascenone	0.020	0.006
**22**	hexanoic acid	0.020	0.060
**23**	2-phenyl ethanol	0.010	0.050
**24**	β -ionone	0.003	0.010

Table S2. Multiple comparison test (Tukey HSD test) results considering the cultivar as independent variable.

Multiple comparisons: Tukey HSD test			
**Dependent Variable**	**Cultivar**	**Cultivar**	***p.***
ethyl hexanoate	Nero di Troia	Aglianico	0.002
		Primitivo	0.001
ethyl decanoate	Nero di Troia	Primitivo	0.028
diethyl succinate	Primitivo	Aglianico	0.015
		Nero di Troia	0.006
ethyl isopentyl succinate	Nero di Troia	Primitivo	0.038
butyric acid	Nero di Troia	Primitivo	0.012
1-hexanol	Primitivo	Aglianico	0.002
		Nero di Troia	0.000
cis-3-hexen-1-ol	Aglianico	Nero di Troia	0.000
		Primitivo	0.000
	Nero di Troia	Primitivo	0.000
linalool	Aglianico	Nero di Troia	0.034

Table S3. Standardized Canonical Discriminant Function Coefficients of 14-months aged Ctr and US VOC profile of wines.

Compound	Function 1
ethyl Acetate	-8.192
3-methyl-1-butyl acetate	2.078
ethyl hexanoate	2.852
ethyl octanoate	1.099
ethyl decanoate	16.619
diethyl succinate	2.720
ethyl isopentyl succinate	-8.168
monoethyl succinate	2.843
acetic acid	-3.408
isobutyric acid	11.847
butyric acid	-18.765
isovaleric acid	.013
1-butanol	-1.325
2+3 methyl Butanol	4.340
1-hexanol	-2.521
